# Sequence analysis of *Plasmodium vivax* Duffy binding proteins reveals the presence of unique haplotypes and diversifying selection in Ethiopian isolates

**DOI:** 10.1186/s12936-021-03843-7

**Published:** 2021-07-10

**Authors:** Lemu Golassa, Alebachew Messele, Eniyou Cheryll Oriero, Alfred Amambua-Ngwa

**Affiliations:** 1 Aklilu Lemma Institute of Pathobiology, Addis Ababa University, Addis Ababa, Ethiopia; 2 Medical Research Council Unit The Gambia At London, School of Hygiene and Tropical Medicin, Banjul, The Gambia

**Keywords:** *Plasmodium vivax*, PvDBPII, DARC, Polymorphism, Mutations

## Abstract

**Background:**

Red blood cell invasion by the *Plasmodium* *vivax* merozoite requires interaction between the Duffy antigen receptor for chemokines (DARC) and the *P. vivax* Duffy-binding protein II (PvDBPII). Given that the disruption of this interaction prevents *P. vivax* blood-stage infection, a PvDBP-based vaccine development has been well recognized. However, the polymorphic nature of PvDBPII prevents a strain transcending immune response and complicates attempts to design a vaccine.

**Methods:**

Twenty-three *P. vivax* clinical isolates collected from three areas of Ethiopia were sequenced at the *pvdbpII* locus. A total of 392 global *pvdbpII* sequences from seven *P. vivax* endemic countries were also retrieved from the NCBI archive for comparative analysis of genetic diversity, departure from neutrality, linkage disequilibrium, genetic differentiation, PvDBP polymorphisms, recombination and population structure of the parasite population. To establish a haplotype relationship a network was constructed using the median joining algorithm.

**Results:**

A total of 110 variable sites were found, of which 44 were parsimony informative. For Ethiopian isolates there were 12 variable sites of which 10 were parsimony informative. These parsimony informative variants resulted in 10 nonsynonymous mutations. The overall haplotype diversity for global isolates was 0.9596; however, the haplotype diversity was 0.874 for Ethiopia. Fst values for genetic revealed Ethiopian isolates were closest to Indian isolates as well as to Sri Lankan and Sudanese isolates but further away from Mexican, Papua New Guinean and South Korean isolates. There was a total of 136 haplotypes from the 415 global isolates included for this study. Haplotype prevalence ranged from 36.76% to 0.7%, from this 74.2% were represented by single parasite isolates. None of the Ethiopian isolates grouped with the Sal I reference haplotype. From the total observed nonsynonymous mutations 13 mapped to experimentally verified epitope sequences. Including 10 non-synonymous mutations from Ethiopia. However, all the polymorphic regions in Ethiopian isolates were located away from DARC, responsible for junction formation.

**Conclusion:**

The results of this study are concurrent with the multivalent vaccine approach to design an effective treatment. However, the presence of novel haplotypes in Ethiopian isolates that were not shared by other global sequences warrant further investigation.

**Supplementary Information:**

The online version contains supplementary material available at 10.1186/s12936-021-03843-7.

## Background

Of the *Plasmodium* species infecting human, *Plasmodium vivax* is responsible for the most geographically widespread form of malaria. Once considered benign, *P. vivax* is now known to cause recurrent infections that can sometimes lead to severe life-threatening pathologies [1, 2]. Though chemotherapies are available, there is as yet a vaccine due to challenges with polymorphisms and the presence of multiple strains in endemic regions that restrict strain-transcending global protection [3–6]. The pathological outcomes of *P. vivax* infection depend on the parasite's ability to identify, invade human erythrocytes and develop clinical levels of parasitaemia. The molecular mechanisms of invasion of immature red blood cells by *P. vivax* requires a Duffy Binding Protein II (PvDBPII) ligand, which interacts with its human receptor on the surface of erythrocytes, the Duffy Antigen Receptor for Chemokines (DARC). As PvDBP-DARC interaction is critical, individuals lacking the Duffy antigen on their erythrocyte surface (Duffy negatives) are resistant or have reduced susceptibility to *P. vivax* infection [7, 8]. Moreover, acquired antibodies against PvDBPII can inhibit PvDBP-DARC interaction and *P. vivax* invasion, partly qualifying PvDBPII as a leading asexual blood stage vaccine candidate against *P. vivax*. However, PvDBPII like other Duffy-binding orthologues, is particularly polymorphic especially at its amino terminal cysteine-rich region II (PvDBPII). This hampers the rational design of potent DBP-based vaccines and necessitates the identification of globally conserved epitopes across parasite populations for evaluation in possible multivalent vaccine designs. Therefore, knowledge of the nature and levels of genetic polymorphisms in PvDBPII among global *P. vivax* isolates is important toward vaccine development.

Analysis of PvDBPII protein sequences identified seven haplotypes represented in 60% of global *P. vivax* populations [9]. African isolates were poorly represented in this data partly due to relative dominance of *Plasmodium falciparum* cases in Africa and mostly Duffy-negative human populations that are resistant to *P. vivax* infection [10]. Though the Duffy blood group antigen (Fy^a^ or Fy^b^) is not expressed on red blood cells (RBCs) of the majority of individuals of African origin [11], low levels of *P. vivax* malaria has now been reported across much of Africa using sensitive molecular diagnosis methods [7, 12–15]. This has raised questions on the expansion of the infection across more African population as *P. falciparum* is targeted and eliminated. This risk is particularly high in populations where transmission of *P. falciparum* and *P. vivax* is sympatric and at significant levels, such as Ethiopia and population across the Sahara. In Ethiopia, *P. vivax* accounts for almost 30% of all malaria cases indicating the great public health significance of this neglected parasite [16]. Future *P. vivax* vaccines based on PvDBPII must, therefore, assess these populations for global efficacy.

In Ethiopia, evidence of *pvdbpII* gene amplification (three and eight copies) in two Duffy-negative individuals has been reported [17]. Worryingly, a study has also shown that parasites with increased *pvdbpII* copy number are able to infect erythrocytes even in the presence of naturally acquired antibodies that would have otherwise blocked binding of PvDBP to DARC [18]. Upon this is antigenic variation, i.e., the ability to produce variable antigens, which enables the parasite to evade host immunity [6]. Thus, a multivalent PvDBPII based vaccine will be most favorable and its design will require knowledge of most strains of *P. vivax* circulating globally. This study describes genetic diversity of *pvdbpII* in isolates from Ethiopia, which is the fourth highest contributor to global vivax infections.

## Methods

### Blood samples, DNA extraction and *P. vivax* diagnosis

A total of 30 *P. vivax* samples were collected in a cross-sectional study conducted from Nov. 2018 to Dec. 2018 in East Shoa Zone, Ethiopia. The blood samples were collected from clinical malaria patients attending health facilities. Thick and thin blood films prepared for species identification. Blood samples were spotted onto filter papers for parasite DNA extraction. Vivax malaria-infected patients were treated by chloroquine. Genomic DNA was extracted from DBS using QIAamp DNA Mini kit (QIAGEN, Hilden, Germany) according to manufacturers’ protocol. DNA extracts were then used to confirm *P. vivax* infections by qPCR using the GENESIG *Plasmodium vivax* standard kit (PrimerDesign Ltd, Chandler’s Ford, UK) according to manufacturers’ protocol. Cycling conditions on the CFX96^TM^ Real-Time PCR detection system (Bio-Rad Laboratories Ltd, California, USA) include enzyme activation at 95 °C for 2 m, followed by 50 cycles of denaturation at 95 °C for 10 s and annealing/data acquisition on the FAM channel at 60 °C for 60 s.

### Amplification and sequencing of the PvDBPII

The PvDBP region was amplified using a modified version of previously published PCR conditions [18] and comparing amplicons from two different forward primers (PvDBP_N1_fwd 5′- GATAAAACTGGGGAGGAAAAAGAT-3′ and PvDBP_N2_fwd 5′- CCTCGAATGGTGGCAATCCT-3′) in combination with a single reverse primer (PvDBP_N1_rev 5′- CTTATCGGATTTGAATTGGTGGC-3′) to give amplicons of approximately 1243 bp and 1062 bp respectively. Final amplification conditions contained 0.02U/µl of Q5® High-Fidelity DNA Polymerase enzyme in 1X Q5® High-Fidelity DNA Polymerase Buffer (New England Biolabs, Hitchin, UK), 0.2 mM dNTPs, 0.1 ng/ml of Bovine serum albumin (BSA), 0.5µM of the forward and reverse primers and 5µl of template DNA. Cycling conditions were 98 °C of enzyme activation for 30 secs, followed by 35 cycles of denaturation at 98 °C for 10 s, annealing at 62 °C (for amplicon 1–1243 bp) or 67 °C (for amplicon 2–1062 bp) for 30 s, elongation at 72 °C for 60 s and a final extension at 72 °C for 120 s. Amplicons were purified using Sephadex G-50 (Merck, Darmstadt, Germany) and purified amplicons were then used as templates for cycle sequencing reaction, using the Applied Biosystems™ BigDye™ Terminator v3.1 Cycle Sequencing Kit, according to manufacturers’ protocols. Sequences were detected on the SeqStudio genetic analyzer (Applied Biosystems, California, USA). *PvdbpII* sequences were successfully obtained for 23 Ethiopian sequences. For each isolate, trace files for *pvdbpII* were curated and assembled by Seqman pro in Lasergene v 15.3 (DNASTAR) to generate consensus sequences. The latter were aligned using ClustalW alignment algorithm in MEGA X. For comparative analysis, a total of 392 global *pvdbpII* sequences from 7 *P. vivax* endemic countries were retrieved from the NCBI archive. These included 100 isolates from India (FJ491142.1-FJ491241.1), 35 from Mexico (KP759780-KP759814), 12 from Myanmar (JN25576.1-JN255587.1), 89 from PNG (AF469515-AF469602), 13 from South Korea (JN989472.1-JN989484.1), 100 from Sri-lanka (GU143914-GU143949), and 43 from Sudan (MG805616-MG805657). The reference sequence from Sal-1 (DQ156512) was included in the final analysis.

### Genetic diversity of *pvdbpII* sequences

DNA sequence polymorphisms and diversity statistics were analysed using DnaSP v 6.12. The following genetic diversity parameters were assessed: number of segregating sites (S), nucleotide diversity (π), haplotype diversity (Hd), average number of pair wise nucleotide differences (K) within the population, and total number of mutations ($\eta$). To test if population diversity at *pvdbpII* deviated from neutrality, Tajimas D and Fu and Lis F indices were calculated for the overall protein region sequenced as well as by scanning across the regions using the sliding window approach (window size of 90 bp and a step size of 3 bp). Departures from neutrality were further assessed using the rates of synonymous (dS) and non-synonymous (dN) mutations, estimated and compared by the Z-test (P < 0.05) in MEGAx using the Nei and Gojobori method with jukes cantor correction and 1000 bootstrap replications.

Linkage disequilibrium (LD), effective population size, probability of recombination between adjacent nucleotides per generation and minimum number of recombination events (Rm) were measured using DnaSP. Genetic differences between populations were determined using Wier and Cockerham’s Fst. To visualize the global haplotype network between the isolates, the median joining algorithm as implemented on NETWORK v10. STRUCTURE v 2.3.4 was used to resolve the genetic structure of *P. vivax* haplotypes. For STRUCTURE, data were run for values K2-K9 using admixture ancestry model, each run had a burn in period of 50,000 iterations and 100,000 Markov chain Monte carlo replications. The most probable K value was then calculated according to Evannos method by using the STRUCTURE harvester website. CLUMPAK was used to graphically display results produced by the STRUCTURE software. To investigate the effects of host immune pressure on the diversity of B and T cell epitopes, the genetic diversity in in-silico predicted and known B and T cell epitopes and MHC-binding regions in PvDBPII were examined. These epitopes were mapped against the tertiary structure of PvDBPII extracted from the Protein data bank (6R2S).and 3RRC which shows crystal structure of region ii from *P. vivax* Duffy binding protein (Dimerization of *P. vivax* DBP is induced upon receptor binding and drives recognition of DARC).

## Results

### High level of diversity in PvDBPII

Out of the 30 samples that tested positive for *P. vivax*, *pvdbpII* was successfully amplified and sequenced from 23 *P. vivax* single infections as verified using qPCR. The 23 sequences ranged from 958 to 1190 bp. Further analysis was constrained to a 675 bp region that aligned across all isolates from Ethiopia and the global source from NCBI (total of 415 sequences). From these, 110 variable sites were detected, of which 44 were parsimony informative. For Ethiopian isolates only, there were 12 variable sites of which 10 were parsimony informative (contains at least two types of nucleotides, or at least two of them occur with a minimum frequency of two). These parsimony informative variants resulted in 10 nonsynonymous mutations. Overall global isolates in this study showed 66 singleton mutations, i.e., observed in only one isolate. The overall haplotype diversity for global isolates was 0.9596, while for Ethiopia it was 0.874. The observed nucleotide diversity for Ethiopian isolates was 0.00608 (Table 1) with highest peaks of nucleotide diversity with the *pvdbpII* coming between the middle intersection of the alignment (196–366) base pairs. Sliding window analysis reveals that nucleotide diversity (π) ranged from 0.014 to 0.019 in this region.

**Table 1:** Nucleotide diversity parameters for *pvdbpII* gene in eight *P. vivax* endemic countries

Countries	No. of isolates	S	H	Hd	K	π
Ethiopia	23	12	9	0.87352	4.08696	0.00608
India	100	36	35	0.92141	5.90222	0.00885
Mexico	35	10	7	0.55294	2.68908	0.00400
Myanmar	12	27	12	1.000	8.25758	0.001229
PNG	89	61	42	0.91267	6.29826	0.00937
S. Korea	13	14	12	0.98718	3.69231	0.00549
Sri Lanka	100	27	39	0.92242	6.54020	0.00973
Sudan	42	17	15	0.89547	4.01742	0.00598

*S *Segregating sites, Hd Haplotype diversity, *K* Average number of pair- wise nucleotide differences, *π* Nucleotide diversity

### Balancing selection signatures map around PvDBPII immune epitopes

**Fig. 1:**
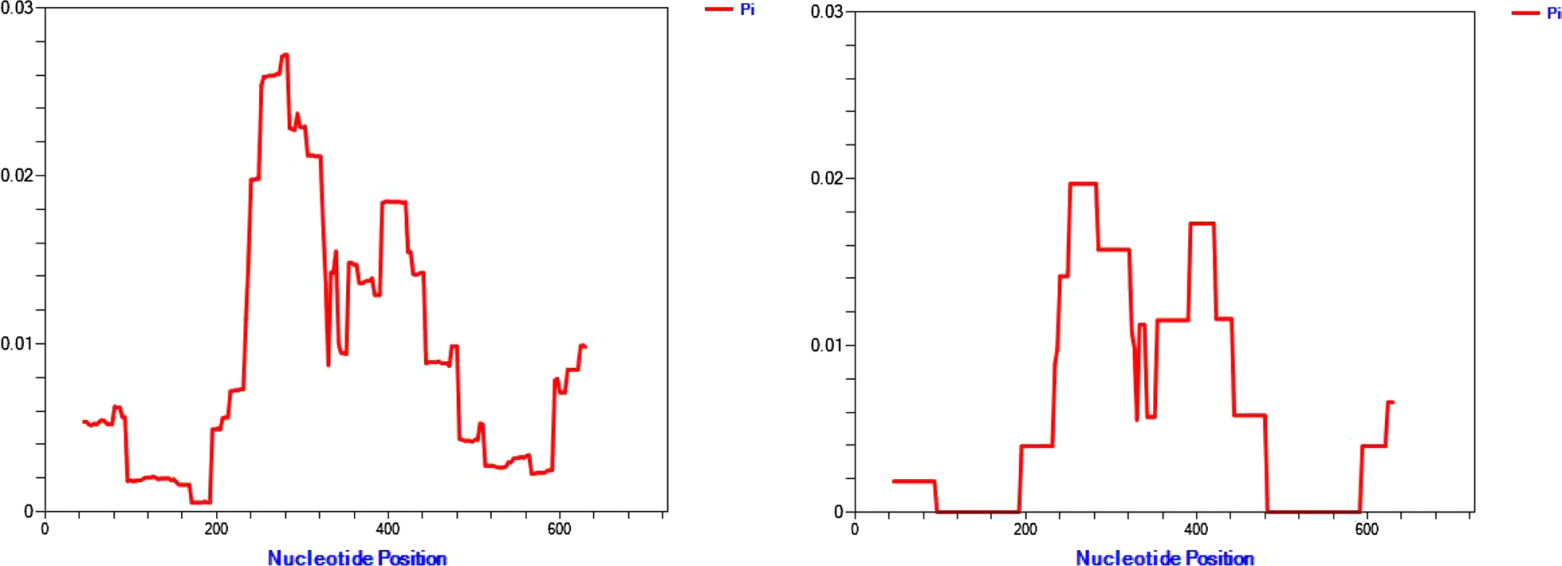
Sliding window analysis (window size 90, step size 3) for Nucleotide diversity (π) in Global (left) and Ethiopian (right) using *pvdbpII* sequence

**Fig. 2:**
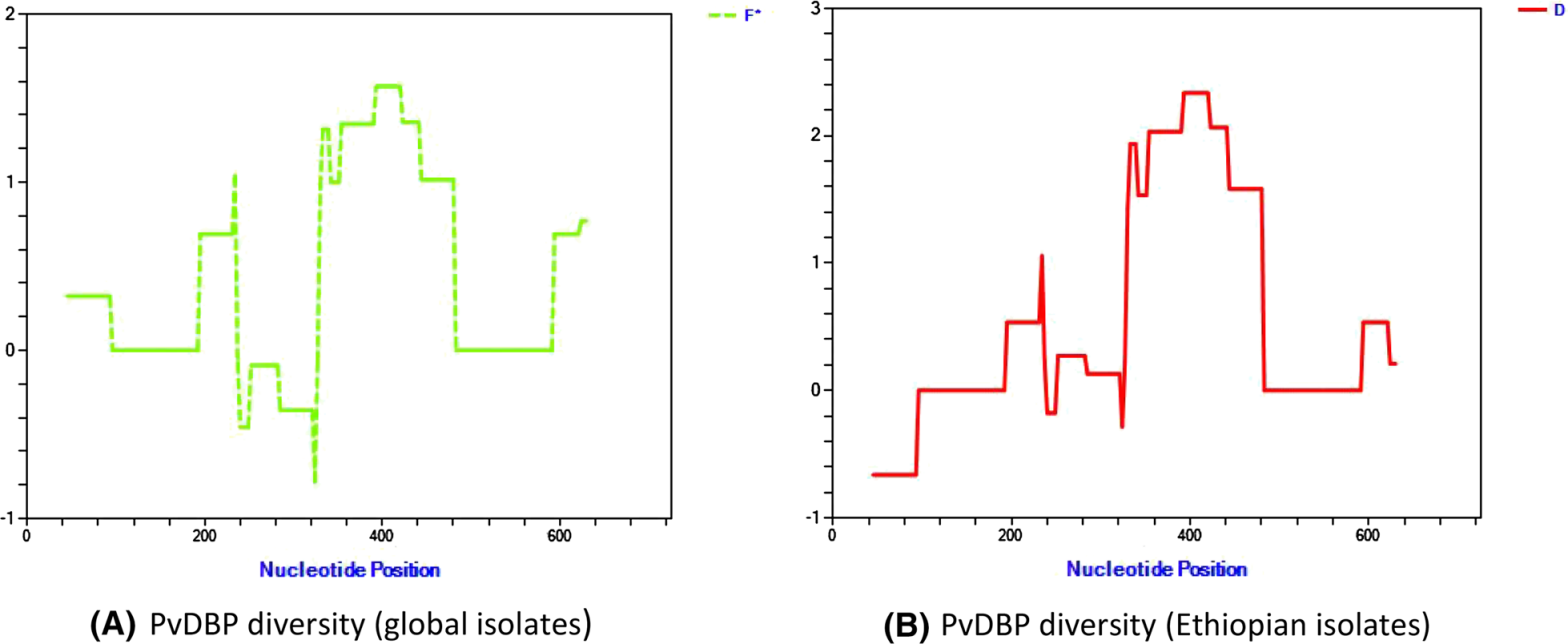
Sliding window analysis (Window size 90, step size 3) results for Departure from Neutrality using Fu and Lis F (left) and Tajimas’ D (right) for Ethiopian *pvdbpII*’ sequences

Overall tests for neutrality by Fu and Lis’ F and Tajimas’ D were positive for *pvdbpII* from all populations, with values of X and Y, respectively for Ethiopian isolates. Sequence diversity was heterogeneous across the region with highest Pi values around nucleotides 200 to 500 (Figs. 1 and  2). Both Fu and Lis D and F tests detected significant positive values in Ethiopian isolates in the region that spans from 265–465 bp (1.5704 P < 0.10). Significant positive values were also observed for Tajimas D value in the region 289–384 (1.9349 P < 0.10) for global isolates and 310–486 (2.0651 P < 0.05) for Ethiopian isolates.

The regions with higher signatures of balancing selection were dominated by non-synonymous mutations. The global set of isolates exhibited 30 nonsynonymous mutations, of which 13 mapped to epitope sequences (Table 2). The observed 10 non-synonymous mutations in Ethiopia equally mapped against regions of balancing selection and mapped/corresponded to experimentally verified epitopes.

**Table 2:** Polymorphic regions (highlighted) that map to known epitopes sequences

Epitope name	Epitope	Sequence
5	T/B	VNNTDTNFH**R**DITFR
45	B	SIFGT**GEK**AQQ**H**RKQ
48	B	**EK**AQQ**H**RWWNESK
66	T	ICK**I**NVAVNIEPQIY
Ia	MHC class I	G**N**FIWICK**I**
Ic	MHC class I	VLSNKF**K**SVKNAEK
IIa	MHC class II	YSVKKRLKG**N**
IIb	MHC class II	FIWICK**I**NY

**Fig. 3:**
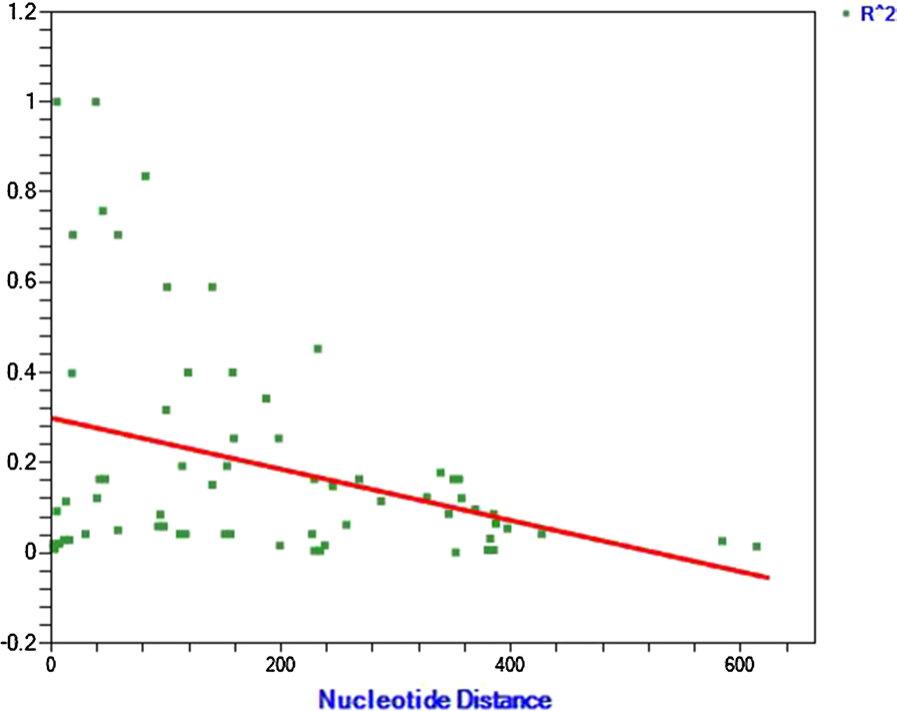
Linkage disequilibrium plot of R^2^ for *pvdbpII* gene from Ethiopian isolates

The source of genetic variation was tested for recombination using the minimum recombination events as implemented in DnaSP. In global *P. vivax* populations the minimum numbers of recombination events between adjacent polymorphic sites (Rm) was high in Indian, Myanmar and Sri Lankan isolates all of which were > 5 (Table 3). In compliment LD index R^2^ declined across analysed region in Ethiopian isolates an indicator of intragenic recombination as a possible means for increased diversity (Fig. 3).

**Table 3:** Estimates of recombination events in *pvdbpII* gene in eight global *P. vivax* endemic countries

Country (No. of isolates)	R^a^	R^b^	Rm
Ethiopia (23)	0.0261	17.5	2
India (100)	0.0240	16.1	10
Mexico (35)	0.000	0.001	1
Myanmar (12)	0.2623	176	7
PNG (89)	0.0146	9.8	3
South Korea (13)	0.0741	49.7	1
Sri Lanka (100)	0.0206	13.8	9
Sudan (42)	0.0310	20.8	3

R^a^ recombinant parameter between adjacent sites, R^b^ recombination parameter for whole gene, Rm minimum number of recombination events

### Differentiation at PvDBPII between populations

Pairwise population Fst values at PvDBPs between the eight *P. vivax* endemic populations ranged from 0.0193 between Sri Lanka and India to 0.3647 between South Korea and Mexico (Table 4). Ethiopian isolates were closer to Indian and Sri Lankan compared to Sudanese isolates despite geographic proximity between Sudan and Ethiopia. They were most diverged from Mexican, Papua New Guinean and South Korean isolates.

**Table 4:** Genetic differentiation (Fst) values of the *pvdbpII* gene among eight *P. vivax* endemic geographical populations

Ethiopia	India	Mexico	Myanmar	PNG	South K	Sri lanka	Sudan
India	0.0422						
Mexico	0.1940	0.2500					
Myanmar	0.0846	0.0499	0.1458				
PNG	0.1275	0.1180	0.2766	0.1314			
South K	0.1301	0.1226	0.3647	0.1441	0.2454		
Sri_lanka	0.0529	0.0193	0.2653	0.0826	0.1302	0.1158	
Sudan	0.0945	0.1328	0.0673	0.0660	0.2239	0.1927	0.1495

**Fig. 4:**
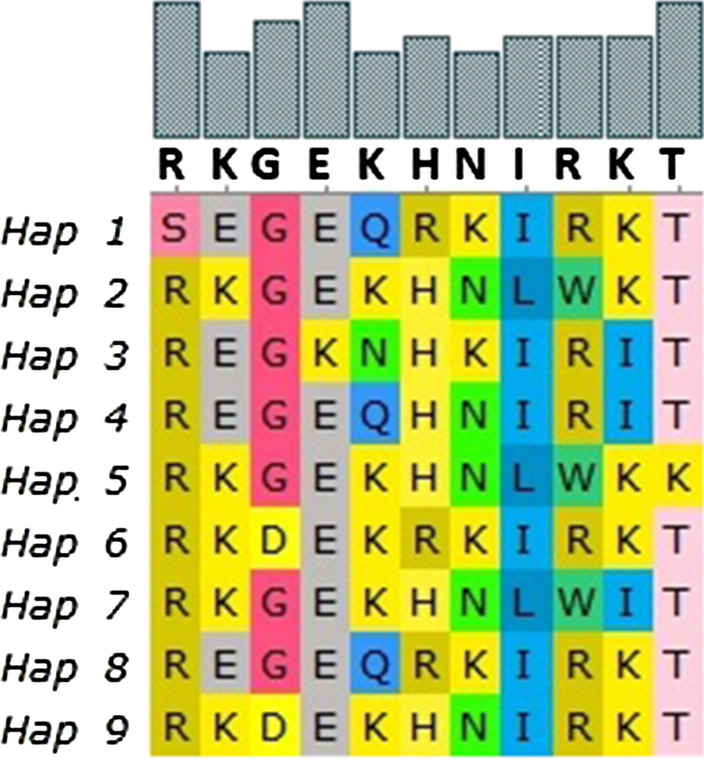
*PvdbpII* haplotypes observed among Ethiopian isolates.*There were nine haplotypes observed from the 23 Ethiopian isolates *(Fig. 4)

**Fig. 5:**
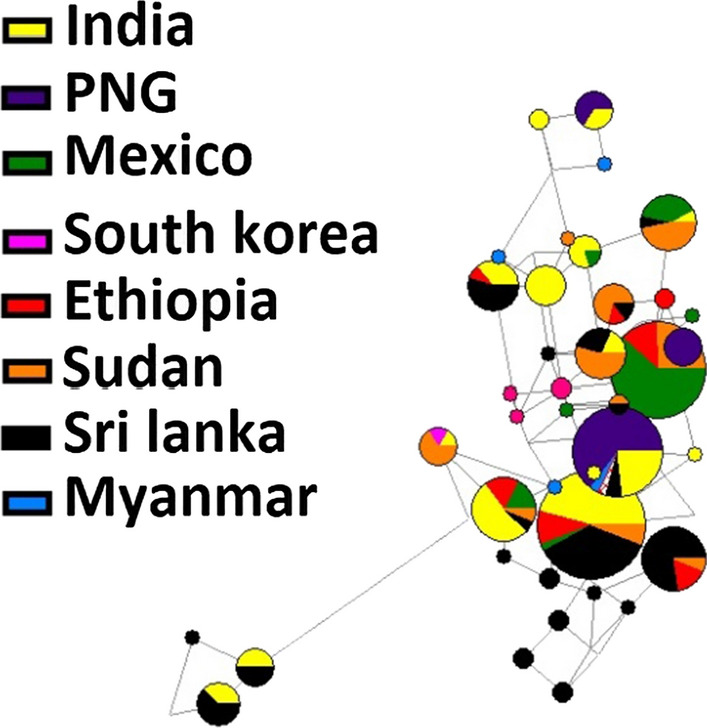
Network analysis of *pvdbpII* haplotypes using the median joining algorithm implemented in Network software. The Pies represent the haplotypes with lines indicating the connections, size of each pie corresponds to frequency, and colors indicate the geographic populations

Nine distinct haplotypes of *pvdbpII* were detected in Ethiopian isolates (Fig. 4). Of the 9 haplotypes, 6 had the highest frequency with 6 isolates followed by haplotypes 2 with 5, haplotypes 5 and 7 had 3 isolates each whereas there were 2 isolates for haplotype 1, while the rest had one isolate each (Fig. 5). There was a total of 136 haplotypes from the 415 global isolates analysed. Haplotype prevalence ranged from 36.76% to 0.7%, with 74.2% represented by a single parasite isolate. None of the Ethiopian isolates grouped with the Sal I reference haplotype; instead two formed their own cluster (1.47%), five clustered together in the dominant haplotype type represented in 50 isolates (36.76%) from India, Sri Lanka and Sudan. Another five Ethiopian isolates grouped in a cluster of 39 isolates from Mexico and Sudan (28.67%), three grouped with 18 isolates from Sri Lanka and Sudan (13.24%), further three grouped with 17 Indian and Sudanese isolates (12.4%), whereas four isolates formed their own unique single haplotype cluster each.

All the polymorphic regions in Ethiopian isolates were located away from the Duffy antigen receptor for chemokines responsible for junction formation (Additional file 1). In sharp contrast the polymorphic region from other isolates showed that a mutation is present in this critical region.

## Discussion

The interaction between the Duffy antigen for chemokine receptors in humans and the Duffy binding protein in *P. vivax* is critical for the subsequent invasion of reticulocytes. This pathway is currently the only known way the parasite colonizes its human host. However, the loss of this receptors in erythrocytes has been shown to reduce susceptibility or complete resistance to *P. vivax* infection [7, 8]. As a result studies have targeted the Duffy binding protein as a potential vaccine candidate, which would be capable of blocking entrance to its host [4]. In support of this hypothesis, several studies have shown that the polymorphic sites of the Duffy binding protein are localized and that most of the sequence remains conserved [9]. This is largely down to the structural biology of the protein and the nature of its junction formation with the Duffy antigen for chemokine receptors [19]. In order to maintain this junction, the protein needs to maintain its structural rigidity as an alteration could lead to a loss of forming this critical network with its receptor [20]. Therefore, much of the region responsible for attachment is highly conserved. Instead, the protein shows polymorphism away from these regions, mostly on B and T cell epitopes or around them. The protein uses these polymorphic sites to exercise antigenic diversity, providing the protein the capability of immune evasion. Therefore, to use this protein as a vaccine, a multivalent vaccine approach is critical, i.e., the ability to cover the diversity of circulating strains globally. However much of these studies have focused on non-African strains, instead targeting only on Asian, South American and Australian strains. This is counterproductive as Africa and particularly Ethiopia is the one of the highest contributors to morbidity rates in the world [16]. Much remains unclear in terms of understanding the nature of the Duffy binding protein in Ethiopia, particularly circulating strains, nature of haplotypes and genetic diversity. Here for the first time, the haplotype diversity of *pvdbpII* and natural selection of Ethiopian PvDBPII and the haplotype prevalence based on predominant, non-synonymous single nucleotide polymorphisms (SNPs) are reported.

There were 10 nonsynonymous mutations in Ethiopian isolates, all of which mapped exclusively to epitopes regions. This was concurrent with observation with other global isolates; however, other isolates exhibited nonsynonymous mutation in other regions as well, albeit near the epitopes. This agrees with the hypothesis that immune pressure from the host is responsible for the diversifying selection occurring on this region. However, the extent of immune selection in Ethiopian isolates seems to be much more pronounced as evidenced from the departure of neutrality test. Indeed, significant positive values would indicate diversifying selection, whereas significantly negative values would indicate purifying selection. Analysis of Ethiopian isolates reveals significant positive values for both Tajimas D and Fu and Lis f tests. Furthermore, both tests showed that selection was operating in the same location as the epitopes, confirming the immune selection hypothesis. This trend was also similar for global sequences although significantly positive values were not found for Ethiopian isolates. In compliment, genetic diversity (π) was highest in two regions particularly that are found in the central domain of the protein. For Ethiopian isolates this ranged from 0.014 to 0.019, a mark of localized diversity. This was also in complement to the departure of neutrality test which found significantly negative values in regions away from this peak diversity. It has been indicated that higher levels of polymorphism may also reflect the artifact of differences in sampling, PCR and/or sequencing, which may have caused South Korean isolates to have higher genetic diversity as compared to countries endemic with high vivax malaria such as Colombia and PNG [21, 22]. However, nucleotide diversity was moderate in Ethiopian isolates in relation to other countries, and only specific regions appeared to have significantly higher nucleotide diversity as such it is not expected to be a result of sampling and/or sequencing artifacts.

Meiotic recombination has previously been indicated as a potential source of genetic variation in *pvdbpII*. Here recombination was evaluated using DnaSP minimum recombination event parameter and as an indirect indicator of Linkage disequilibrium. In the event of meiotic recombination, the frequency of linkage disequilibrium declines significantly with increasing distance. Indeed, linkage disequilibrium showed significant decline in the analysed region suggesting a role for recombination. Similarly, DnaSP found regions that were recombining, although to a lesser extent in Ethiopian and South Korean isolates. It has been suggested that this could be a result of a declining *P. vivax* transmission or an indication of malaria intervention (could be related to vivax seasonality or transmission strength) [22]. However other global population showed higher recombination values indicating the contribution of Recombination towards genetic diversity. This is in agreement with several other studies, which examined the effects of recombination in *P. vivax* endemic countries. As such it is also likely that the test used to detect recombination for Ethiopian isolates may not have sufficient power to detect low recombination events. Low transmission can explain why recombination is low while balancing selection maintains the already high genetic diversity observed in Ethiopia, indeed samples were taken from low transmission setting and hence might explain why genetic diversity is high while recombination is low.

Fst index was used to assess the degree of population differentiation and gene flow in the eight *P. vivax* endemic countries. Ethiopian isolates showed lower degree of differentiation with Indian isolates, perhaps unsurprisingly given the expanding *P. vivax* population and/or extensive gene flow supported by intense human migration between the two countries. On the other hand, little differentiation was also seen in Ethiopian and Sri Lankan isolates, Ethiopian and Sudanese isolates. It is also likely that the strong selection pressure resulted in low Fst values, as fixation of certain alleles may also affect the result [5]. Moreover, relatively high degree of differentiation was exhibited between Ethiopian and Mexican, Ethiopian and Papua New Guinean, Ethiopian and South Korean isolates. This could be due to the geographic barrier between the countries inhibiting gene flow. Indeed, Fst values exceeded 0.25 which would refer to highly differentiated populations, in isolates from India PNG and South Korea vs Mexican isolates showing a reduced gene flow. Similarly, it is quite possible that the strong selection pressure exerted in this gene may have caused a fixation of alleles in certain geographic regions while remaining absent in others. It should be noted that Fst values are affected by factors such as population structure, where the test assumes a stable population structure which may not be the case for *P. vivax* infections. However other studies have indeed found population clustering in relation to geographic origin and distance, as confirmed using AMOVA and STRUCTURE analysis, similarly the same held true for Ethiopian isolates using STRUCTURE analysis.

High numbers of haplotypes were found in the Ethiopian datasets, interestingly, this was like a recent global *pvdbpII* haplotype analysis study which previously identified seven haplotypes that can cover 60% parasite population in 8 malaria endemic countries [9]. However, the previous study used isolates from non-African countries and recommended an inclusion of representative haplotypes. Accordingly, few studies have explored genetic diversity of *pvdbpII* in African countries, such as Sudan. The study in Sudanese isolates found that only one unique haplotype was not included in global haplotype diversity [23]. The finding of this study show four unique haplotypes in the Ethiopian isolates that were not shared with other global isolates, perhaps indicating that there are haplotypes that have not been considered for vaccine trials. Indeed, the frequency of this haplotypes in this study in was not significant as three of the four only had one representative. Even so the low number of samples in this study compared to the number of haplotypes observed, will warrant further study. As such further studies on the frequency of circulating haplotypes in Ethiopia will give a clear indication as to which are the dominant strains that need be included for global vaccine trials.

Haplotype diversity was indeed high in most of the population included in this study, although it is much more pronounced in Sri Lankan and Indian isolates. In the Sri Lankan isolates haplotype diversity reach 1, showing the extent of variability in the population. In Ethiopia, however, haplotype diversity was intermediate in relation to the other included population sets. Indeed, again a small number of samples included could have limited the power of the analysis, but even so the analysis found nine types of haplotypes from 23 Ethiopian isolates. Five of these haplotypes were part of the major haplotype cluster responsible for 36.7% of the global population included for this study. A total of 136 haplotypes were results of singleton mutations, hence the results support the previously mentioned study which identified seven haplotypes that can cover 60% parasite population in 8 malaria endemic countries [9].

Subsequently this region was mapped to a recently experimentally validated tertiary structure of PvDBPII [20]. This structure was determined using a reliable X-ray diffraction method, as opposed to a previous homology modelled structure. This was advantageous as homology modelling is not as reliable as x ray diffraction. This may have affected a 2010 study which found a high genetic diversity and residues under strong positive selection near the DARC receptor domain [24]. This would result in the blockage of junction formation, virtually inhibiting red cell invasion. Apart from this, the study stipulated that antibodies involved in invasion inhibitory activity also block PvDBP-DARC interaction. However recent studies on *pvdbpII* copy number amplification have shown that they are able to infect even in the presence of naturally acquired antibodies [18]. This would at least indicate the presence of other mechanism for immune evasion such as antigenic variation. This is also supported by subsequent worldwide studies, which have shown many mutations to be predominantly placed on epitopes which agreed with the results of this study [5, 9]. This result is further supported by departure of neutrality tests and now a reliable tertiary structure of PvDBPII which placed non-synonymous mutations away from the DARC recognition site.

## Conclusion

This study provides the first description on genetic diversity; the prevalence and nature of *pvdbpII* sequence polymorphisms in Ethiopian field *P. vivax* isolates. The results of this study support the multivalent vaccine approach to design an effective treatment. However, the presence of novel haplotypes that were not shared by other global sequences warrant further investigation. As such additional studies need to be made to assess *pvdbpII* sequence polymorphism by collecting large number of clinical isolates from various heterogenous regions of Ethiopia. This will be an important step in designing and developing an efficacious PvDBPII vaccine capable of covering global strains.

## Supplementary Information

**Additional file 1.** 3-D structure of *P. vivax* Duffy binding protein bound to human antibody and its polymorphic sites.

## Data Availability

Not applicable.

## Abbreviations

DARC: Duffy antigen receptor for chemokines
Hd: Haplotype diversity
K: Average number of pair wise nucleotide differences within the population
LD: Linkage disequilibrium
$\eta$: Total number of mutations
π: Nucleotide diversity
SNP: Single nucleotide polymorphism
S: Segregating sites
dS: Rates of synonymous
dN: Rates of non-synonymous mutations
PCR: Polymerase chain reaction
PvDBPII: *Plasmodium vivax* Duffy binding protein II
RBCs: Red blood cells
