# Supplementary material for: Sequence analysis of Plasmodium vivax Duffy binding proteins reveals the presence of unique haplotypes and diversifying selection in Ethiopian isolates
Source: Malar J. 2021 Jul 10;20:312. doi: 10.1186/s12936-021-03843-7 (PMC8271342; doi:10.1186/s12936-021-03843-7)
Supplement: Supplementary file 1 — Additional file 1. 3-D structure of P. vivax Duffy binding protein bound to human antibody and its polymorphic sites. [file 12936_2021_3843_MOESM1_ESM.doc]

**Supplementary matwerials**


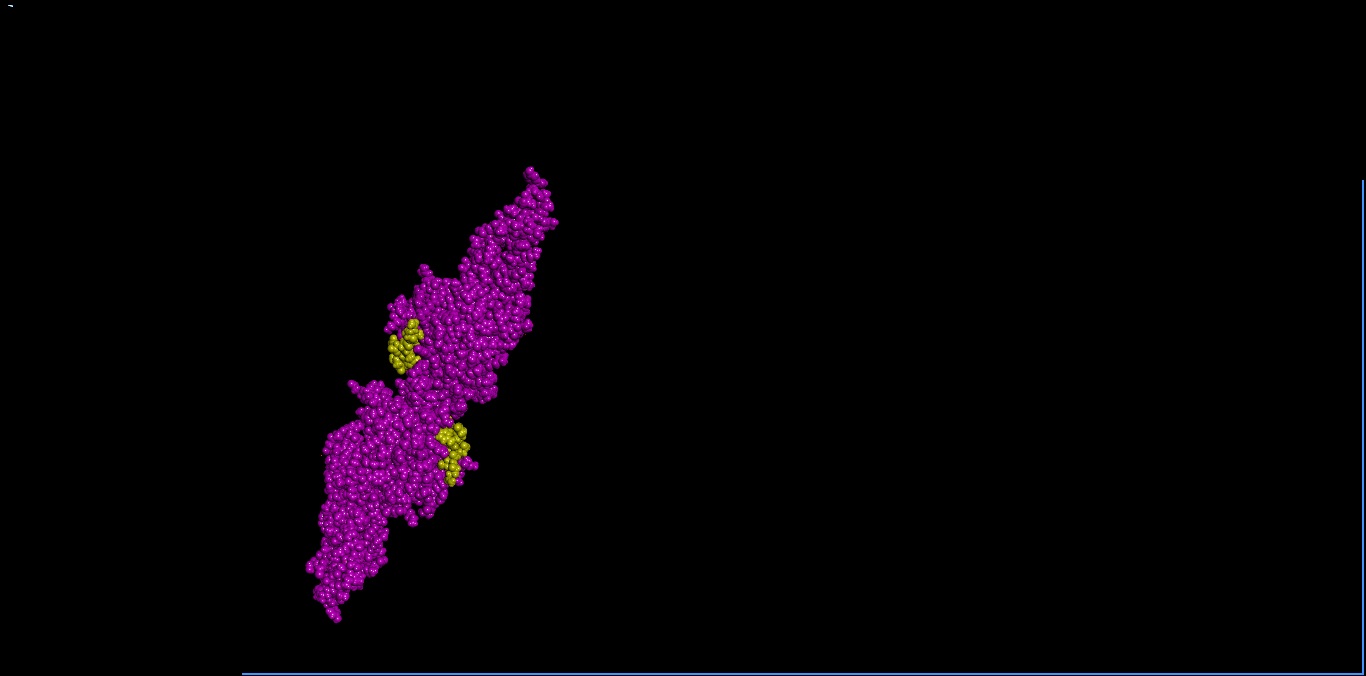


1. DARC binding site


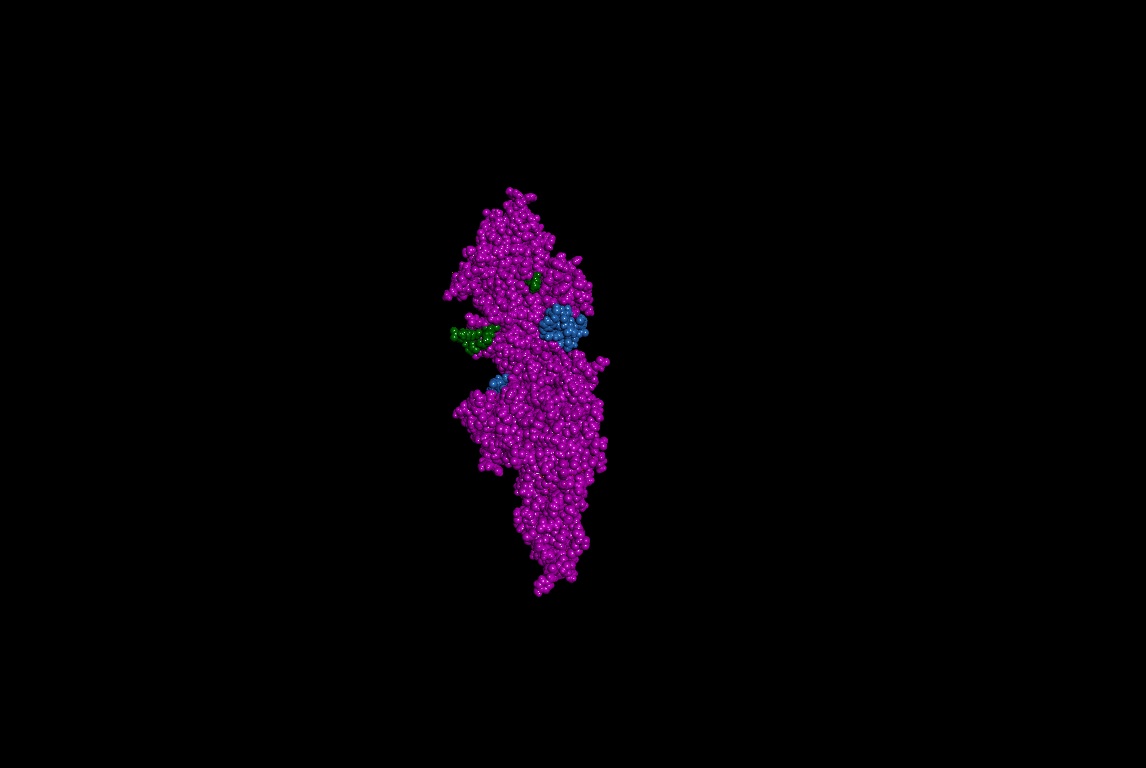


2. Binding site and polymorphic epitope


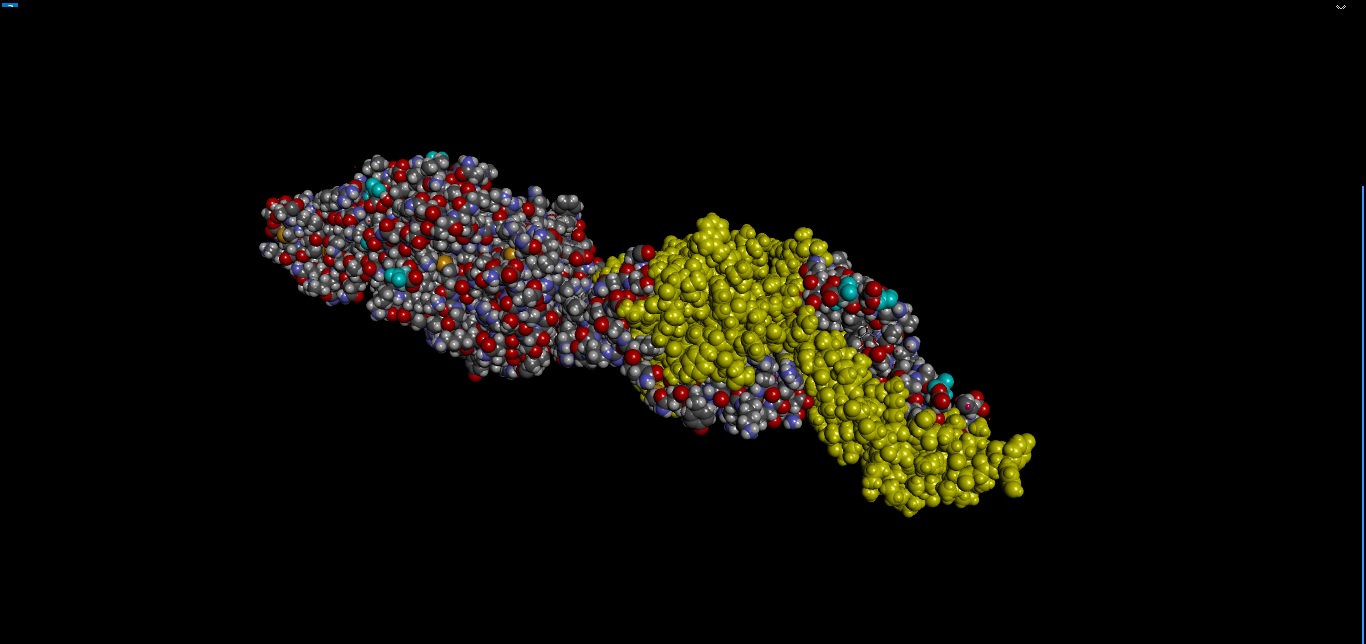


3.675 bp regions included for analysis
